# Variant RONΔ160 of the RON receptor tyrosine kinase promotes the growth and invasion in vitro and in vivo in gastric cancer cell lines

**DOI:** 10.1186/s12935-015-0157-5

**Published:** 2015-02-04

**Authors:** Dong-Hui Zhou, Chao Li, Li-Na Yang

**Affiliations:** Department of Oncology, First Affiliated Hospital, Zhejiang University School of Medicine, Hangzhou, 310003 China; Department of Oncology, Dongnan Affiliated Hospital of Xiamen University, Zhangzhou, China

**Keywords:** Gastric cancer, RON, RONΔ160, MSP, Tumorigenicity

## Abstract

**Background:**

Recepteur d’origine nantais (RON) is a receptor tyrosine kinase whose overexpression has been observed in human gastric cancers. This study aimed to determine whether overexpression of the variant RONΔ160 could induce tumorigenicity of gastric cancer cells in vitro or in vivo, and whether its specific small molecule inhibitor (Compound I) could inhibit the effect of RONΔ160.

**Methods:**

We constructed human gastric cancer cell line MGC-803 that was stably transfected with a recombinant plasmid expressing RONΔ160, and the effect of RONΔ160 overexpression and macrophage-stimulating protein (MSP) activation on proliferation, migration and invasion abilities of MGC-803 cells were evaluated. Tumor-bearing mice with gastric cancer cells were used to analyze the effects of RONΔ160 overexpression and Compound I on implanted tumor growth.

**Results:**

In vitro, overexpression of RONΔ160 in MGC-803 cells resulted changes to their cell morphology, and promoted cell proliferation, migration and invasion. In addition, overexpression of RONΔ160 increased the proportion of cells in the S phase. The effect of RONΔ160 was significantly enhanced by induction of MSP inducing (*p* < 0.05). In vivo, RONΔ160 promoted the growth of MGC-803 cells in nude mice, including increased tumor size and weight, and lower tumor incubation period. The Compound I inhibited the tumorigenic abilities of RONΔ160 (*p* <0.05).

**Conclusions:**

The results indicate that overexpression of the variant RONΔ160 altered the phenotype and tumorigenicity of MGC-803 cells. Its specific small molecule inhibitor could inhibit the effect of RONΔ160. Therefore, the variant RONΔ160 may become a potential therapeutic target for gastric cancer.

## Background

Gastric cancer is one of the most common malignant tumors, whose onset is difficult to diagnose. At the time of definite diagnosis, most patients are at the middle or late stage, and their response therapy and prognosis are poor. Thus, it is especially important to identify new molecular markers and therapeutic targets for gastric cancer. Studies have shown that the abnormal expression of RON, a member of the large RTK protein family, plays an important role in the incidence and development of tumors [1]. RON also plays an important role in the growth, differentiation and proliferation of epithelial cells. RON is barely or not expressed in normal epithelial tissue, while in breast, colon, lung, thyroid, skin, bladder and pancreas tumors it is highly expressed and is usually accompanied by the generation of variants [2,3]. Currently, nine types of RON variants have been identified from primary carcinoma and cell lines, which are termed RONΔ170, RONΔ165, RONΔ165.e11p, RONΔ160, RON^E5/6in^, RONΔ155, RONΔp110, RONΔ85 and RONΔ55, respectively [4-7]. These variants are generated by three main mechanisms: alternative splicing, protein truncation and alternative transcription of precursor RON mRNA [8]. RON is activated mainly in a ligand dependent manner. Currently, the sole known ligand of RON is macrophage-stimulating protein (MSP). Maturate MSP is formed by the binding of the 53 kDa α chain with the 30 kDa β chain through disulfide bonds. The binding of RON with the β chain significantly increases RON’s kinase activity and results in phosphorylation of the kinase domain [9]. RON contains functional domains that play important roles in ligand binding, protein maturation and bioactivity [5,10]. For example, deletion of the first immunoglobulin-plexin-transcription (IPT) unit of the extracellular segment of β chain produces variant RONΔ160, which is oncogenic and mediates many signals for tumor generation [11]. However, the effect of RONΔ160 on the incidence and development of gastric cancer has not been reported.

In this study, we explored the impact of RONΔ160 on the growth and invasion of MGC-803 cells by producing a derivative of MGC-803 that stably expresses high levels of RONΔ160. We also investigated the inhibitory action of a small molecule inhibitor of RONΔ160. This established the theoretical basis for exploring new therapeutic targets for the gastric cancer.

## Materials and methods

### Materials

Human gastric cancer line MGC-803 was obtained from the State Key Laboratory for Diagnosis and Treatment of Infectious Diseases, The First Affiliated Hospital, Zhejiang University School of Medicine. PrimeScript™ RT reagent kit and SYBR Premix Ex Taq™ reagent kits were purchased from Takara Biotechnology (Dalian Co., Ltd, China). Lipofectamine™ 2000 and the cell cycle reagent kit were purchased from Invitrogen (Life Technologies, USA). Hygromycin B and c-Met/RON small molecular inhibitor (Compound I) were obtained from Calbiochem. Recombinant human MSP was purchased from R&D systems. Cell counting kit 8 (CCK-8) was purchased from Dojindo Molecular Technologies (Rockville, USA). Antibodies against RON (β-chain) were purchased from Santa Cruz Biotechnology (USA). The recombinant plasmid pcDNA3.1-RONΔ160 and blank plasmid pcDNA3.1 were obtained from Sangon Biotech Co., Ltd (China). Female Balb/c athymic nude mice (Nu/Nu) aged 6-weeks were obtained from the B&K Universal Group and were kept in temperature-controlled rooms with a 12-h alternating light-dark cycle.

### Cell cultures and transfection

MGC-803 cells were cultured in RMPI-1640 medium with 10% fetal calf serum (FCS). One day before transfection, cells were plated at a density of 60%-70% confluence. Transfection was made in the following groups: MGC-803 cells transfected pcDNA3.1-RONΔ160 (RONΔ160 group), MGC-803 cells transfected with blank plasmid pcDNA3.1 (empty vector control group), and non-transfected MGC-803 cells (MGC-803 group). Transfection was conducted in accordance with the instruction of Lipofectamine™ 2000 reagent. After 24 hours, the cells were digested and diluted (1:10) with fresh culture media. After a further 24 hours 200 mg/L of Hygromycin B was added to select positive clones, which were obtained after about 2 weeks. Positive clones were expanded in culture medium with 200 mg/L Hygromycin B. Ultimately, MGC-803 cells stably expressing pcDNA3.1-RONΔ160 and blank plasmid pcDNA3.1 were obtained after about 8 weeks.

### Western blotting

Proteins from each group were separated through 8% SDS-PAGE and then transferred to polyvinylidene difluoride (PVDF) membranes. The membranes were blocked with 5% nonfat milk for 2 h at room temperature and incubated with specific antibodies overnight at 4°C. After thorough washing, the membranes were incubated with HRP-conjugated secondary antibody. Visualization of the reaction was conducted with enhanced chemiluminescence (ECL) reagents and analyzed by VersaDoc Imaging system (Bio-Rad).

### Quantitative real-time RT-PCR

According to the manufacturer’s instructions, the PCR reaction was carried out with the following primers: RONΔ160 forward: 5′-CTTGGCTGAGGTCAAGGATGTG-3′; RONΔ160 reverse: 5′-CGATTCTGGGCCTGGTCTATG-3′; β-actin forward: 5′-CCAACCGCGAGAAGATGA-3′; β-actin reverse: 5′-CCAGAGGCGTACAGGGATAG-3′. The amplification profile comprised precycling at 95°C for 20 s; followed by 40 cycles of denaturation at 95°C for 15 s, annealing at 60°C for 60 s and extension at 72°C for 40 s.

### CCK-8 assay

Cells were seeded into 96-well plates at a concentration of 2 × 10^3^ cells/well, which were cultured in RPMI 1640 medium with 1% FCS. A RONΔ160 + MSP group of cells was created, to which were added MSP of final concentration 2nM. After seeding for 24-168 hours, the proportion of live cells was detected consecutively using CCK-8 reagent. Finally, the absorbance (OD) value was measured with a microplate reader at wavelength of 450 nm. The experiment was carried out in triplicate.

### Transwell invasion assay

The cells were seeded at a density of 5 × 10^4^ cells/chamber into the Transwell upper chamber (8.0 μm size micro-pore filtering membrane). The lower chamber was filled with 600 μl of 10% FCS containing RPMI 1640 culture media. After 24 h of incubation, the cells were fixed for 30 minutes with methanol. After washing with PBS, the cells that penetrated to the lower surface of the filter were stained with 0.1% crystal violet solution and counted under the microscope at a magnification × 400 field.

### Wound healing assay

Cells from each group were seeded into 6-well plates at concentration of 5 × 10^5^ cells/well, and cultured in 1% FCS-containing RPMI 1640 culture media. At 95% confluence, the wounds were created with 200 μl micropipette tip. MSP at a final concentration of 2 nM was added into the RONΔ160 + MSP group for further stimulation. The cells were photographed at 0 hour and 48 hours after wounding, and the percentages of cell migration distance in 48 hours compared with the blank scratching distance at 0 hour were calculated.

### Cell cycle assay

After digestion with trypsin, 2 × 10^5^–1 × 10^6^ cells were collected, washed with PBS, fixed in pre-cooled 70% ethanol overnight at -20°C., The ethanol was removed after centrifugation, the cells were hydrated with PBS for 15 minutes, before 50 μl RNase A was added and the cells incubated in a 37°C water bath for 30 minutes. Finally, 200 μl of propidium iodide was added to cells for 30 minutes at 4°C with light-shielding to stain the cells. The cells were analyzed by flow cytometry. (FACScan, Calif., USA).

### In vivo tumor growth and treatment

The effect of RONΔ160 on the tumorigenic ability of MGC-803 cells was determined by analyzing the subcutaneous tumor formation ability in 6-week-old BALB/c athymic female nude mice. The backs of the nude mice were subcutaneously planted with cells and the experiment was conducted in groups as follows: CP-I group was subcutaneously planted with MGC-803 cells stably transfected with RONΔ160, and from the second day they were intraperitoneally injected with the small molecule inhibitor c-Met/RON (Compound I) at dosage of 100 mg/kg for 4 weeks, twice every week; the RONΔ160 group was subcutaneously planted with MGC-803 cells stably transfected with pcDNA3.1-RONΔ160; the MGC-803 group was subcutaneously planted with MGC-803 cells; and control group was subcutaneously planted with MGC-803 cells transfected with the blank plasmid pcDNA3.1. The latter three groups were injected with normal saline using the same protocol the same as that for the CP-I group. The growth of subcutaneous-transplanted tumors was observed daily. After the formation of tumors, the tumor volume was measured every 3 days, and the incubation period of the tumor was recorded, i.e. the time from transplantation of tumor cells till the day when the tumor reached a volume of 500 mm^3^. After the tumor cells had been implanted for 40 days, the nude mice were killed by cervical disruption, the tumors were peeled off, and their volumes and weights were measured. All experiments involving mice were carried out using the recommendations of the Guide for the Care and Use of Laboratory Animals of the National Institutes of Health and were approved by the Zhejiang University Animal Care and Use Committee.

### Statistical analysis

Data were summarized as mean ± standard error ($\overline{X}\pm S$). Statistical significance was determined by Student’s *t* test or by ANOVA, using SPSS 19.0 software. A P-value less than 0.05 was considered to be statistically significant.

## Results

### Recombinant plasmid pcDNA3.1-RONΔ160 stably transfected into MGC-803 cells induces morphological changes

**Figure 1:**
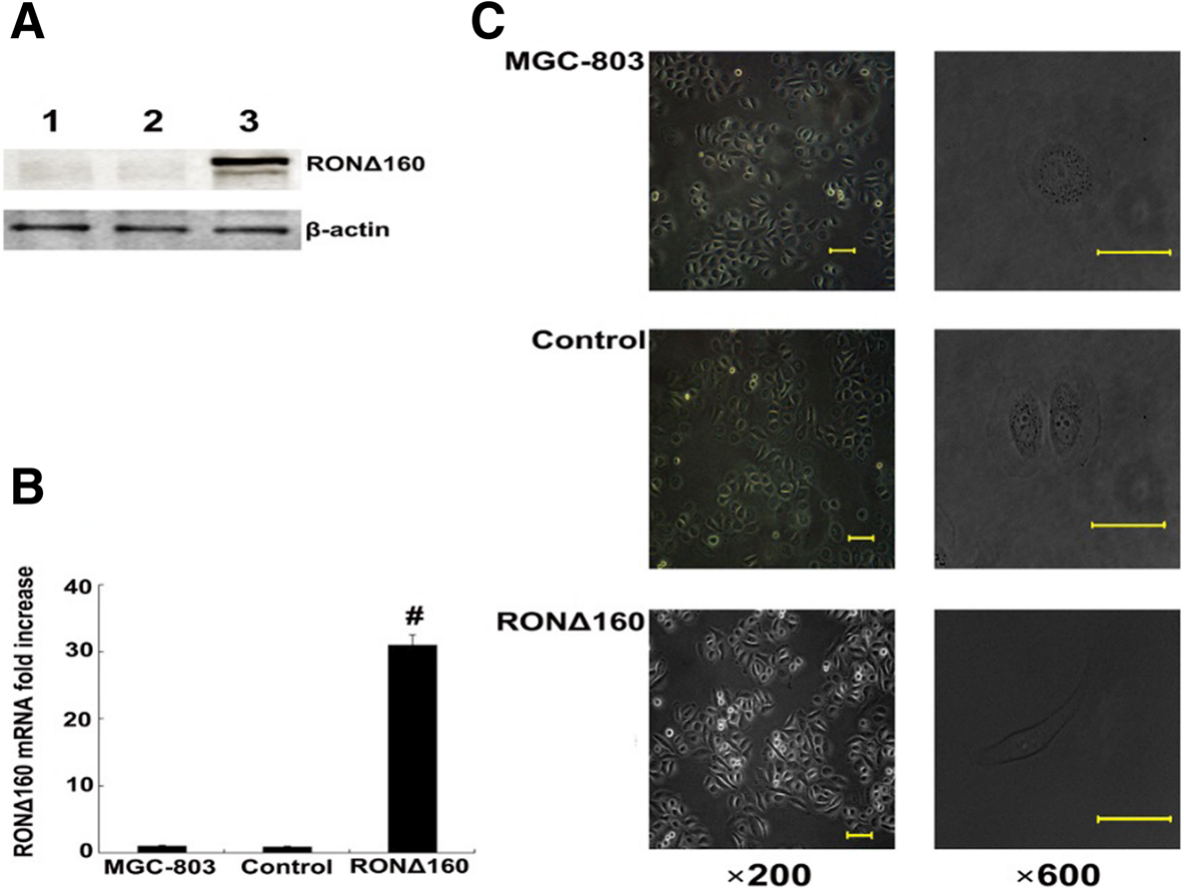
**RONΔ160 is stably transfected into MGC-803 cell line and induces a change in morphology. A)** Western blotting results: Lane 1, MGC-803 Group; Lane 2, empty vector control (control); Lane 3, RONΔ160 Group. **B)** Real-time RT-PCR result: β-actin mRNA was used as an internal control. #: *p* <0.01. **C)** RONΔ160 induces a change in morphology from previous flat circular shape or polygon, to a spindle.

After screening MGC-803 cells for transfection with the recombinant plasmid pcDNA3.1-RONΔ160, western blotting and real-time quantitative PCR verified that pcDNA3.1-RONΔ160 was successfully transfected into MGC-803 cells and expressed. The RONΔ160 protein and mRNA expression levels of the RONΔ160 group cells were significantly higher than those of the control and MGC-803 group (p < 0.01). The empty vector control and MGC-803 group showed almost no expression (Figure 1A,B). The cell morphology of the RONΔ160 group changed from the previous flat circular shape or polygon to a spindle shape (Figure 1C).

### RONΔ160 improves the proliferation of MGC-803 cells

**Figure 2:**
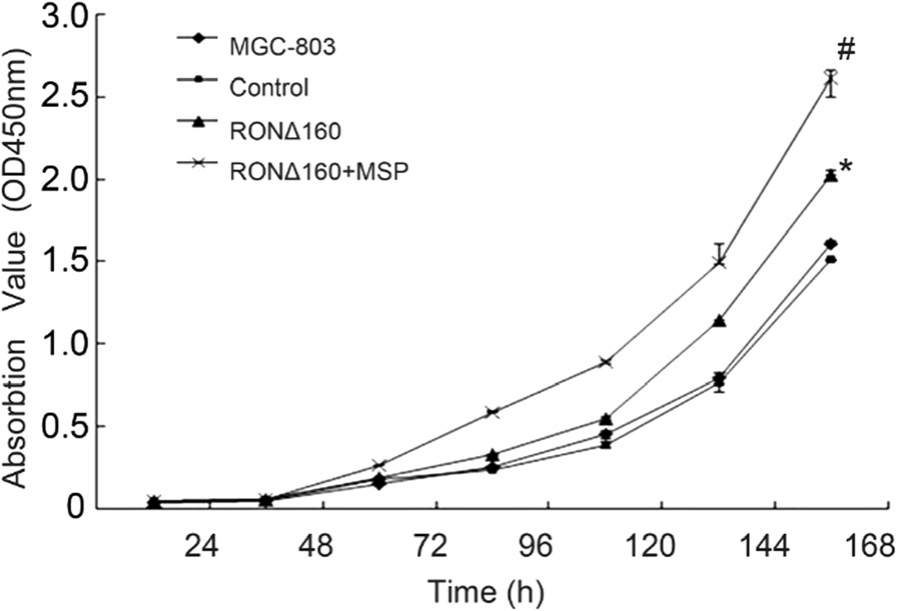
**RONΔ160 improves the proliferation of MGC-803 cells, and MSP increases this effect. #**: *p* < 0.05 (compared with the MGC-803 Group, empty vector control and RONΔ160 Group); *:*p* < 0.05 (compared with the MGC-803 Group and empty vector control).

Cell proliferation was measured with the CCK-8 cell proliferation assay. The results showed that the overexpression of RONΔ160 promoted significant proliferation of MGC-803 cells in the RONΔ160 group compared with the MGC-803 and empty vector control (*p* < 0.05). Meanwhile, the cell proliferation in cells overexpressing RONΔ160 and stimulated with MSP (the RONΔ160 + MSP group) was significantly higher than in the RONΔ160 group, MGC-803 group and empty vector control (*p* < 0.05) (Figure 2).

### RONΔ160 increases the invasive capacity of MGC-803 cells

**Figure 3:**
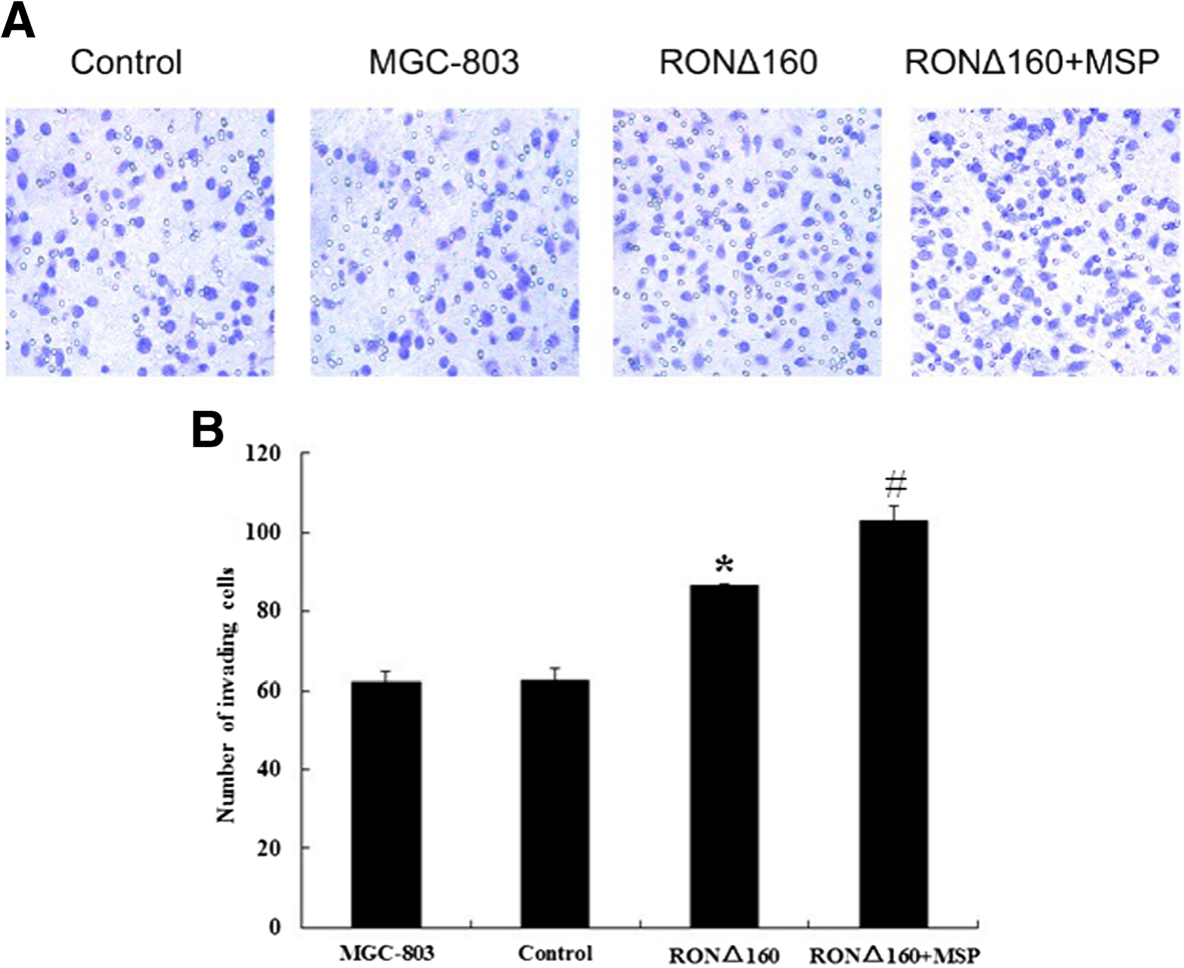
**RONΔ160 increases the invasive capacity of the MGC-803 cell line (×400). A**: Compared with MGC-803 group (61.95 ± 2.78), empty vector control (62.35 ± 3.32) and RONΔ160 group (86.48 ± 0.32), *p* < 0.05; compared with RONΔ160 group (86.48 ± 0.32) and RONΔ160 + MSP group (103.03 ± 3.69), *p* < 0.05; **B**: *: Compared with MGC-803 group, empty vector control, *p* < 0.05; #: Compared with MGC-803 group, empty vector control and RONΔ160 group, *p* < 0.05.

Cell invasion capacity was reflected by the number of cells that penetrated the bottom chamber of the Transwell chamber through the artificial substrate Matrigel that simulates the cell outer basement membrane. The results showed that the invasive capacity of MGC-803 cells of the RONΔ160 group (86.48 ± 0.32) was significantly higher than that of MGC-803 group (61.95 ± 2.78) and the empty vector control (62.35 ± 3.32) (*p* < 0.05). Meanwhile, the cell invasive capacity of the RONΔ160 + MSP group (103.03 ± 3.69) was significantly higher than the RONΔ160 group (*p* < 0.05) (Figure 3A,B).

### RONΔ160 promotes the motility of MGC-803 cells

**Figure 4:**
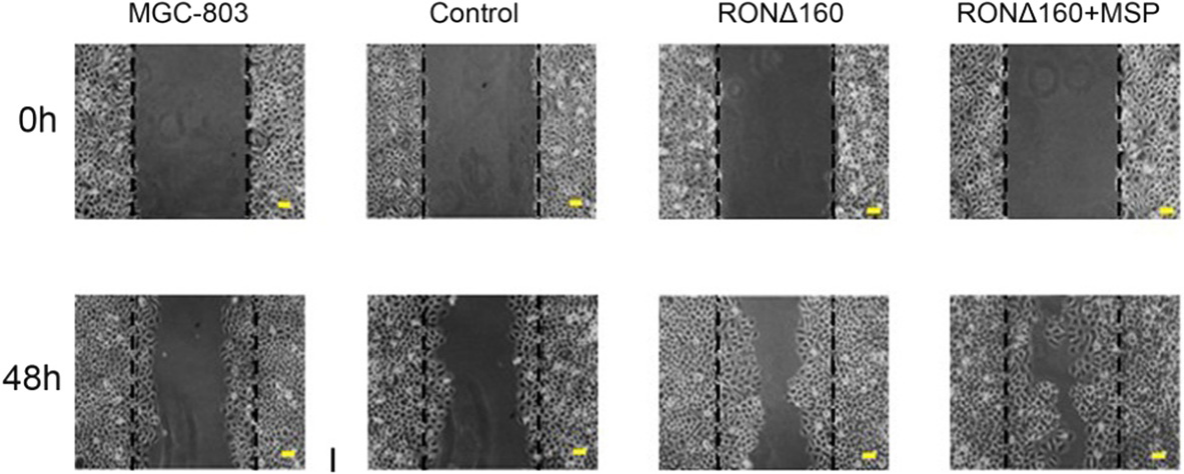
**RONΔ160 increases the motility of MGC-803 cells (×100).** Compared with MGC-803 group, empty vector control and RONΔ160 group, *p* < 0.05; Compared with MGC-803 group, empty vector control and RONΔ160 + MSP group, *p* < 0.05; Compared with MGC-803 group and empty vector control, *p* > 0.05.

The results of the wound healing assay showed that 48 hours after scratching, the migration capacity of cells of the RONΔ160 group (50.41 ± 2.09%) was significantly higher than that of the MGC-803 group (28.70 ± 1.21%) and the empty vector control (26.43 ± 1.69%) (*p* < 0.05). The cell migration capacity of the RONΔ160 + MSP group was significantly enhanced compared with the RONΔ160 (71.18 ± 2.74%) (*p* < 0.05) (Figure 4).

### RONΔ160 regulates the cell cycle activities of MGC-803 cells

**Figure 5:**
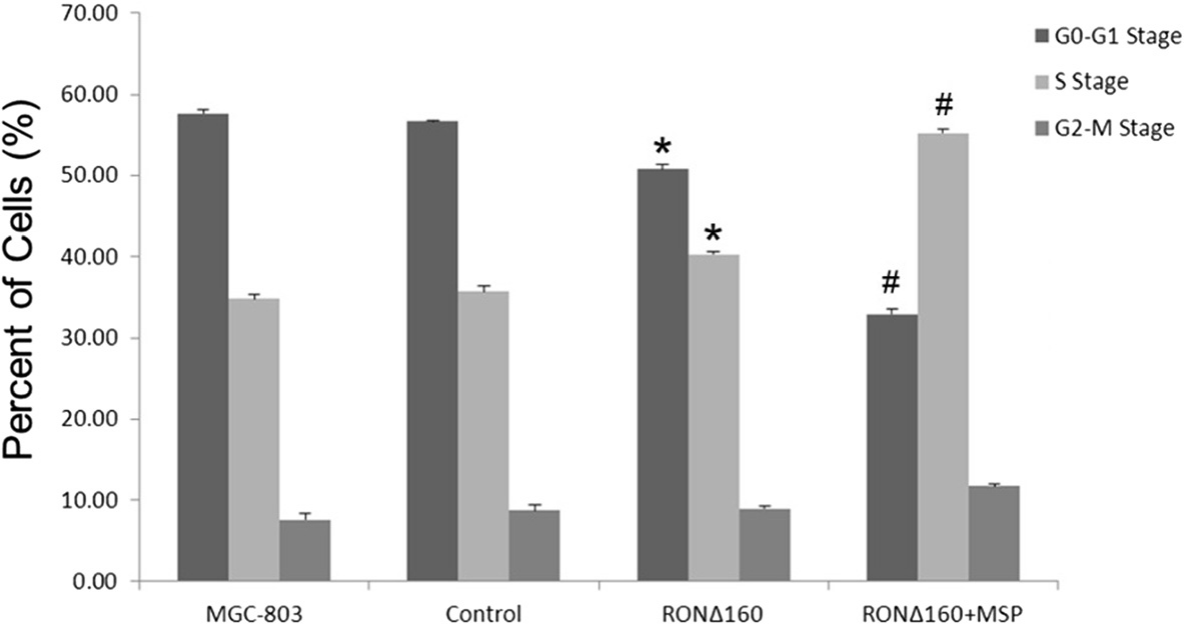
**RONΔ160 results in an arrest in the S phase of the cell cycle in MGC-803 cells.** *: *p* < 0.05 (compared with the MGC-803 Group and empty vector control); #: *p* < 0.05 (compared with the MGC-803 Group, empty vector control and RONΔ160 Group).

The results showed that the proportions of cells in each phase of the cell cycle in the MGC-803 group, empty vector control, RONΔ160 group and RONΔ160 + MSP group were as follows: G0-G1 phase (57.63 ± 0.50%), (56.65 ± 0.22%), (50.78 ± 0.59%), and (32.91 ± 0.66%); S phase (34.77 ± 0.61%), (35.67 ± 0.78%), (40.33 ± 0.25%), and (55.22 ± 0.59%); and G2-M phase (7.61 ± 0.73%), (8.69 ± 0.80%), (8.90 ± 0.35%), and (11.72 ± 0.30%). Compared with the MGC-803 group and the empty vector control, the proportions of S phase cells of RONΔ160 group and RONΔ160 + MSP group were larger, the proportions of cells in G0-G1 phase were relatively smaller, and the proportions of cells in G2-M phase were not significantly different. There were significantly more cells in S and G0-G1 phases in the RONΔ160 + MSP group (*p* < 0.05). The proportions of cells in each phase between the MGC-803 group and the empty vector control were not significantly different (*p* > 0.05). The above results showed that, in terms of the cell cycle, overexpression of RONΔ160 promoted proliferation of MGC-803 cells (Figure 5).

### Overexpression of RONΔ160 promotes tumor growth in vivo and its specific small molecule inhibitor can inhibit tumor growth

**Figure 6:**
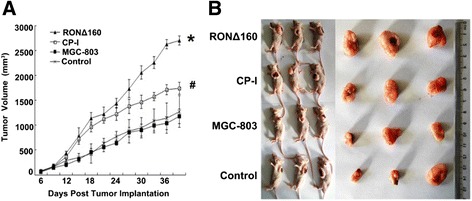
**In vivo effects of RONΔ160 and its specific inhibitor Compound I on MGC-803 cells. A)**, *: *p* < 0.05 (compared with the MGC-803 Group and **empty vector control**); #: *p* < 0.05 (compared with the RONΔ160 Group). **B)**, after implanting tumor cells subcutaneously for 40 days, the nude mice were killed by cervical dislocation, and the tumors were peeled off and photographed.

We further explored the influence of RONΔ160 on tumor growth of MGC-803 cells in tumor-bearing nude mice. The results showed that compared with the MGC-803 group and empty vector control, the tumorigenic ability of cells of the RONΔ160 group was significantly enhanced in vivo by promoting tumor growth, shortening the incubation period of tumor formation and increasing the volume and weight of the tumors. In contrast, compared with the RONΔ160 group, the CP-I group injected with small molecule inhibitor c-Met/RON (Compound I) showed the inhibition of the tumorigenic superiority conferred by RONΔ160 in vivo (p < 0.05) (Table 1, Figure 6).

**Table 1:** **The effects of RONΔ160 and c-Met/RON small cellular inhibitor on MGC-803 cells tumor-bearing nude mice**

	RONΔ160 Group*	**CP-I Group** ^**#**^	**MGC-803 Group**	**Empty vector control**
Tumor volume (mm^3^)	2703.79 ± 92.66	1743.77 ± 119.75	1176.36 ± 149.13	1273.17 ± 316.23
Tumor weight (g)	1.93 ± 0.11	1.21 ± 0.33	0.89 ± 0.29	0.91 ± 0.41
Tumor incubation period (days)	12 ± 1	14.5 ± 1.5	20 ± 1.5	19.7 ± 2.52

*: *p* < 0.05 (compared with MGC-803 Group, empty vector control); ^#^: *p* < 0.05 (compared with RONΔ160 Group).

## Discussion

RON is a receptor with unique tyrosine kinase activity and is a member of the proto-oncogene c-Met family. RON may be activated by ligand-dependent or non ligand-dependent methods. Currently, MSP, which is synthesized by liver cells, is the only known specific ligand of RON. In our previous study, we showed that the expression ratio of RON in gastric cancer tissues was 56.1%, and the corresponding expression ratio in adjacent tissues was 25.6%, whereas there is no or little expression in the normal gastric mucosa. The expression level of RON is directly related to tumor invasion, lymph node metastasis and pathological (TNM) stages, which implies that RON plays an important role in tumorigenesis and development of gastric cancer [12]. Similarly, Song et al. found that the expression of RON in gastric cancer and lymph node metastasis tissue is higher than that in normal gastric mucosa and lymph node tissue without metastasis [13]. The study results of Catenacci et al. showed that RON is overexpressed in 74% of cancer specimens from the gastroesophageal junction; thus, they demonstrated that RON was an important prognostic factor and a potential biological therapeutic target [14]. Hence, RON plays an important role in the pathogenesis of gastric cancer.

Generally speaking, overexpression of RON is accompanied by the generation of variants. Most of the variants can promote the formation of tumors and increase invasion capacity. Studies have shown that the generation of variants plays an important role in maintaining diversified functions of proteins [15]. Some of the RON variants have antagonistic activity to wild-type RON. Other variants, for example RONΔ170, which lacks 46 amino acids encoded by the 19^th^ exon in the kinase domain, is unable to transmit a signal and inhibit formation of tumor [16]. However, most RON variants activate various cell signaling cascades through different substrate specificities and phosphorylation modes, thereby altering cell migration, invasion and proliferation, and promoting the formation of an invasive phenotype and tumor development, e.g. RONΔ160 [2,17]. RONΔ160 is formed by a lack of exons 5 and 6, which encode 109 amino acids of the first IPT unit in the extracellular domain of the RON β chain. RONΔ160 is constitutively activated by phosphorylation of residual groups of tyrosine and can withstand anti-RON antibody-mediated receptor internalization, which contributes to its maintenance of the in-cell signaling cascades and promotes the in vitro transformation of cells and the in vivo growth of tumors [5,11].

We found there was almost no expression of RON and its variant RONΔ160 in the human gastric cancer cell line MGC-803, which makes it a good in vitro carrier for studying the effect of RON on the bioactivity of gastric cancer cells. Through stable-transfection of MGC-803 cells with a variant RONΔ160 expressing plasmid, the overexpression of RONΔ160 in gastric cancer cells was successfully simulated. The observed change in the shape of RONΔ160 overexpressing MGC-803 cells from the previous flat circular shape or polygon to a spindle is similar to the result of Xu et al. [18], which confirmed that overexpression of RON changes the original shape of MDCK cells. The change of cell shape is likely related to RONΔ160-mediated epithelial-mesenchymal transition (EMT), i.e. losing the normal characteristics of epithelial cells and manifesting a malignant transformed mesenchymal cell phenotype [6]. In addition, the cell proliferation, migration and invasion assays showed that overexpression of RONΔ160 significantly promotes proliferation, migration and invasion of MGC-803 cells in vitro. These effects were significantly enhanced after stimulation with RON’s sole specific ligand MSP, which further confirmed the tumorigenicity of variant RONΔ160. Meanwhile, cell cycle analysis also showed that RONΔ160 expressing cells are significantly more likely to be in the S phase and the proportion in the G0-G1 phase was relatively decreased, which further verified that the overexpression of RONΔ160 promotes proliferation of MGC-803 cells. To further explore the biological function of RONΔ160, the nude mice tumor-bearing animal model was used. The results showed that the in vivo tumorigenic ability of MGC-803 cells stably transfected with the variant RONΔ160 plasmid is significantly increased via promotion of tumor growth, shortening of the incubation period of tumor formation and by increasing the volume and weight of the tumors. Addition of the small molecule inhibitor Compound I inhibited the tumorigenic effects of RONΔ160 and slowed the growth of the transplanted tumors in the nude mice. The RON/c-Met dual kinase inhibitor Compound I specifically interrupted the signal transduction pathways that are mediated by the hepatocyte growth factor and macrophage-stimulating protein, and reduces cell migration in a dosage-dependent way, both in vitro and in vivo [19]. The in vivo experiment conducted by Yao et al. showed that a RON monoclonal antibody could inhibit the activity of RONΔ160, which inhibits the growth of colon cancer cells and has a synergistic antitumor effect with cytotoxic drugs [20]. The above in vivo results further confirmed the tumorigenicity of RONΔ160 and indicate the possibility of using RON as the new therapeutic target for gastric cancer.

In summary, this is the first report of the influence of variant RONΔ160 on the biological behavior of gastric cancer. The overexpression of variant RONΔ160 promotes the proliferation, migration and invasive capacity of a human gastric cancer cell line and its specific small molecule inhibitor can reverse the effects, possibly representing a novel route of targeted therapy. Therefore, our results provide a theoretical basis for the identifying new therapeutic targets for gastric cancer and the development of corresponding targeted drugs.
